# Intra-genomic variation in symbiotic dinoflagellates: recent divergence or recombination between lineages?

**DOI:** 10.1186/s12862-015-0325-1

**Published:** 2015-03-14

**Authors:** Shaun P Wilkinson, Paul L Fisher, Madeleine JH van Oppen, Simon K Davy

**Affiliations:** School of Biological Sciences, Victoria University of Wellington, Kelburn Parade, Wellington, 6012 New Zealand; Australian Institute of Marine Science, PMB No. 3, Townsville, QL 4810 Australia

**Keywords:** Coral, *Symbiodinium*, Symbiosis, *Pocillopora damicornis*, ITS2, Concerted evolution, Sexual reproduction, Recombination

## Abstract

**Background:**

The symbiosis between corals and the dinoflagellate alga *Symbiodinium* is essential for the development and survival of coral reefs. Yet this fragile association is highly vulnerable to environmental disturbance. A coral’s ability to tolerate temperature stress depends on the fitness of its resident symbionts, whose thermal optima vary extensively between lineages. However, the *in hospite* population genetic structure of *Symbiodinium* is poorly understood and mostly based on analysis of bulk DNA extracted from thousands to millions of cells. Using quantitative single-cell PCR, we enumerated DNA polymorphisms in the symbionts of the reef-building coral *Pocillopora damicornis*, and applied a model selection approach to explore the potential for recombination between coexisting *Symbiodinium* populations.

**Results:**

Two distinct *Symbiodinium* ITS2 sequences (denoted C100 and C109) were retrieved from all *P. damicornis* colonies analysed. However, the symbiont assemblage consisted of three distinct *Symbiodinium* populations: cells featuring pure arrays of ITS2 type C109, near-homogeneous cells of type C100 (with trace ITS2 copies of type C109), and those with co-dominant C100 and C109 ITS2 repeats. The symbiont consortia of some colonies consisted almost entirely of these putative C100 × C109 recombinants.

**Conclusions:**

Our results are consistent with the occurrence of sexual recombination between *Symbiodinium* types C100 and C109. While the multiple-copy nature of the ITS2 dictates that the observed pattern of intra-genomic co-dominance may be a result of incomplete concerted evolution of intra-genomic polymorphisms, this is a less likely explanation given the occurrence of homogeneous cells of the C109 type. Conclusive evidence for inter-lineage recombination and introgression in this genus will require either direct observational evidence or a single-cell genotyping approach targeting multiple, single-copy loci.

**Electronic supplementary material:**

The online version of this article (doi:10.1186/s12862-015-0325-1) contains supplementary material, which is available to authorized users.

## Background

The ecological success of scleractinian corals arises from their mutualistic symbiosis with the dinoflagellate alga *Symbiodinium*. Energy-rich compounds provided by the phototrophic endosymbiont enhance coral growth and enable reef development in nutrient-poor tropical oceans (reviewed in [1]). The *Symbiodinium* genus constitutes a genetically diverse assemblage [2], with several clades and sub-clades (types) showing different physiological and ecological characteristics [3-5]. Of particular relevance, differences in symbiont thermal optima are conferred to the host in the form of resistance or susceptibility to coral bleaching [3,5,6], a condition responsible for several large-scale episodes of coral mortality [7-9]. Surviving colonies may acclimatize to warming conditions by replacing thermally-sensitive symbionts with more robust types (‘adaptive bleaching’ or ‘symbiont shuffling’; [3,6,10-14]). However the modified consortium may be unstable [11] and less mutualistic [15]. Furthermore, host-symbiont coevolution fosters strong fidelity between symbiotic partners [16] and hence this strategy appears to be confined to a subset of ‘symbiont flexible’ coral taxa [17]. Finally, symbiont shuffling appears to offer a maximum increase in thermal tolerance of around 1–1.5°C [6]. Given that recent model simulations predict a 1.5-3°C increase by the year 2050 [18], adaptive bleaching will unlikely mitigate the environmental stress that corals are expected to face in the near future. As such, the ‘macro-evolutionary’ potential of the coral symbiosis will likely play a defining role in determining the future of coral reef ecosystems in a warming climate.

Adaptation may occur in *Symbiodinium* through selection acting on both existing genetic variation [19,20] and new genetic variation arising through somatic mutations [21] and/or genetic recombination. *Symbiodinium* is generally considered a predominantly asexual, permanent haploid lineage that diversifies through host-specialization and geographic isolation [19]; however several lines of molecular evidence suggest that cryptic meiotic recombination occurs in this genus. Incongruence between isoenzyme phylogenies and those constructed from both RAPD [22,23] and ITS2 sequence variation [24] implicate allelic recombination, consistent with criteria outlined to distinguish between clonal and sexual eukaryote populations [25]. More recently, meiotic recombination has been inferred from linkage disequilibrium between microsatellite loci [26-30], indicating that extensive shuffling of alleles has occurred within several *Symbiodinium* lineages. Additionally, a recent meta-genomic analysis revealed the presence of six meiosis-specific and 25 meiosis-related functional genes in published *Symbiodinium* genomes [31], providing further evidence that the loss of sexual reproduction has not occurred in this genus.

Morphological similarities among symbiotic dinoflagellates highlight the need to use appropriate genetic tools when addressing the incidence of recombination in this group. Several population- and ‘species’-level markers are currently available, including polymorphic microsatellite loci (e.g. [26-29]), low-copy nuclear genes such as *actin* (e.g. [32]), and mitochondrial and chloroplast sequences (e.g. [33-35]). Yet each of these has drawbacks, such as low taxonomic resolution or a lack of universal primer-binding sequences. The internal transcribed spacer 2 (ITS2) region of nuclear ribosomal DNA (rDNA) is currently the most well-characterised and commonly-used marker in *Symbiodinium* systematics [36]. This is primarily due to its high taxonomic resolution [37-39] and ease of PCR amplification (due to high copy numbers and conserved adjacent sequences). Recent genome-wide pyrosequencing has confirmed the taxonomic utility of the ITS2 region, with the dominant sequence variant offering 97% discrimination efficiency across a range of taxa, and rare intra-genomic variants further aiding species identification [40,41]. Emerging patterns of ITS2 secondary structure promise even higher taxonomic resolving power [41], leading some authors to support its candidacy as a barcode marker for delineating species boundaries in plants and algae [42,43]. Unlike alternative mitochondrial- and chloroplast-encoded sequences and single-copy nuclear genes, the ITS2 region is also bi-parentally inherited, and hence the intra-genomic coexistence of polymorphic sequence variants can reveal the occurrence of recombination [44,45]. However, the multiple-copy nature of rDNA renders it subject to intra-genomic variation arising from a variety of other processes, including the generation of paralogous somatic mutations and the degeneration of functional genes into pseudo-genes [46]. Establishing whether a given ribotype is taxonomically meaningful requires the analysis of individuals rather than multi-genomic samples, necessitating a single-cell approach for unicellular dinoflagellates [20,47]. Despite its obvious advantage in distinguishing between intra- and inter-genomic sequence variation [48,49], single-cell PCR (scPCR) has not been widely used in *Symbiodinium* systematics. This is primarily due to its time-consuming nature, and the difficult task of disrupting the recalcitrant cell wall to extract the nucleic acids. A lack of suitable methodology for isolating, extracting and sequencing DNA from individual *Symbiodinium* cells has meant that intra-genomic variation in this genus has remained virtually unexplored [50]. Fluorogenic-probe based qPCR analysis now offers sufficient sensitivity to quantify ITS2 variants at the sub-clade level, and has been used to quantify polymorphic ribotypes within the individual dinoflagellate genome through the use of a PCR pre-amplification step (nested qPCR; e.g. [51]).

In this study, we developed: (a) a single-cell isolation and DNA extraction protocol for *Symbiodinium*; (b) a single-cell PCR-DGGE method to screen for *Symbiodinium* individuals with additive ITS2 repeats; (c) a nested PCR-qPCR assay to quantify intra-genomic ITS2 sequence polymorphisms within individual cells; and (d) a statistical framework to identify admixture in *Symbiodinium* populations based on proportions of ITS2 sequence variants within the genome. The model selection criterion developed in (d) was then employed to test whether the *P. damicornis* symbiont consortium consists of a single clonal population of symbionts featuring a non-diagnostic polymorphism (NDP); two populations of divergent, homogeneous symbionts; or a mixture of genetically homogeneous symbionts and heterogeneous cells, representing putative inter-lineage recombinants.

## Methods

### Study species and location

This study was carried out at the world’s southernmost coral reef at Lord Howe Island (LHI; Australia). This isolated 14.5 km^2^ volcanic remnant is located around 600 km east of the Australian mainland, and some 200 km to the south of the Elizabeth and Middleton Reefs Marine National Park Reserve. The LHI reef hosts at least 83 species of scleractinian coral, (many of which are endemic; [52]), and a correspondingly diverse and endemic *Symbiodinium* assemblage [53]. The host species investigated was the widely-distributed coral *Pocillopora damicornis*, a thermally-sensitive but fast-growing coral that forms a dominant component of many Indo-Pacific reefs (including LHI; [54]). *P. damicornis* is hermaphroditic and shows an unusual dual-reproductive mode, with the majority of offspring consisting of brooded asexual larvae, complimented by the cryptic simultaneous broadcast-spawning of sexual gametes [55]. This species shows a predominantly sexual reproductive mode at LHI, where it occasionally undergoes intergeneric hybridization with *Stylophora pistillata* [56]. This may arise from suboptimal abiotic conditions selecting for ‘extreme’ hybrid phenotypes and/or a low availability of conspecific gametes [57]. *P. damicornis* transmits symbionts vertically from parent to offspring, and can form a symbiosis with a wide range of genetically and physiologically distinct *Symbiodinium* taxa. In Australian waters alone, *P. damicornis* is found in association with *S. goreauii*, *S. glynni*, *S. trenchii*, and numerous other types lacking formal species description including C1b, C1c, C1c-ff, C1h, C1j, C33, C33a, C42, C42a, C42b, C100, C103, C118, C125 and C126 [4,36,53,58-62]. Of these, *P. damicornis* colonies have been reported as hosting *Symbiodinium* C100, C103 and C118 at LHI [53].

### Sample collection and DNA isolation

Coral sampling was carried out in March 2012 at North Bay (−31.521, 159.047) and Ned’s Beach (−31.513, 159.069), Lord Howe Island, Australia. Three *P. damicornis* colonies were sampled from each site by divers either snorkelling (North Bay; depth 1–3 m) or using SCUBA (Ned’s Beach; depth 14-16 m). Three small branch tips (~1 cm^3^) were taken from each colony using diagonal pliers, and preserved in DMSO preservation buffer (20% DMSO, 250 mM EDTA, NaCl saturated, pH 8.0; [63]). Coral samples were stored at −20°C prior to DNA analysis. A 0.12 cm^2^ area of tissue was removed from the skeleton in 1.5 ml of 0.22 μm filtered seawater (FSW), delivered at high velocity through a circular stencil. A 10 μl sub-sample was taken and centrifuged at 16,100 × *g* for 5 min to pellet the *Symbiodinium* fraction. The supernatant was discarded and the pellet re-suspended in 100 μl DNA buffer (DNAB; 0.4 M NaCl, 50 mM EDTA, pH 8.0). Individual cells (*n* = 30 from each colony) were hand-picked under a light microscope using a heat-elongated glass micro-pipette. Each cell was washed three times in 2 μl DNAB, transferred to a 1.7 ml micro-centrifuge tube with 50 mg acid-washed glass beads (710–1180 μm; Sigma-Aldrich), and milled for 1 min at 50 Hz (Qiagen TissueLyser LT; Qiagen, Valencia, CA, USA) to disrupt the cell wall and release the nucleic acids. TE buffer (10 mM Tris–HCl; 1 mM EDTA; pH = 8.0) was then added to a final volume of 20 μl. For each colony, the extraction process was carried out with the symbiont cell omitted (but with coral tissue homogenate included), to ensure that only intracellular DNA contributed to the PCR amplification signal.

### End-point PCR, DGGE and DNA sequencing

Single-cell DNA template solutions generally contained insufficient DNA for direct PCR-DGGE and qPCR analysis. The partial *nr5.8S*, ITS2 and partial *nr28S* regions were therefore pre-amplified using a shortened end-point PCR protocol, with the outer primers ITSintfor2 [64] and ITS2Rev2 [65]. Thermal cycling included an initial denaturation step of 3 min at 95°C followed by 24 cycles of 15 seconds at 95°C, 15 seconds at 56°C and 10 seconds at 72°C (carried out using an Applied Biosystems Veriti thermo-cycler). Each reaction contained 10 μl of DNA template solution, 1× MyTaq PCR reaction mix (Bioline, Randolph, MA, USA), 15 pmol each primer, and deionised sterile water to a total volume of 25 μl. A template-free control reaction was included with each run.

Pre-amplified PCR products were diluted 1:10^3^ (North Bay colonies) or 1:10^4^ (Ned’s Beach colonies) in deionised sterile water prior to PCR-DGGE and qPCR analysis (these differences were due to shortages of DNA template solutions from the Ned’s Beach colonies, which were used for the initial assay development and optimization process). PCR amplification for DGGE was carried out using the primers ITSintfor2 and ITS2CLAMP [64]. Cycling conditions were as described above, except that an additional 16 thermal cycles were run (40 in total). PCR products (20 μl) were loaded on 200 × 200 × 0.75 mm, 8% denaturing polyacrylamide gels (25-50% denaturant gradient), and run in 1 × TAE at 150 V for 7 h at 60°C (DCode system; BioRad, Hercules, CA, USA) alongside known ITS2 sequences of *Symbiodinium* C100 and C109. Following electrophoresis, gels were stained with ethidium bromide and viewed on a UV trans-illuminator (FirstLight UVP, San Gabriel, CA, USA). Five representative bands at each position were excised, milled for 1 min at 50 Hz with 50 mg glass beads and 200 μl TE buffer, and re-amplified with both clamped and non-clamped primers [64]. DGGE was carried out on clamped PCR products to ensure a single band migrated to the identical position from where it was excised. Corresponding non-clamped products were cleaned with ExoSAP-IT (USB Corporation, Cleveland, OH, USA), and sequenced by the Macrogen Sequencing Service (Macrogen Inc., Seoul, South Korea). Sequences were manually checked and aligned in Geneious v 7.0 (Biomatters Ltd., Auckland, New Zealand) and a BLAST search was carried out against *Symbiodinium* ITS2 sequences available in GenBank. Novel sequences were assigned alphanumeric ITS2 nomenclature (c.f. [24,64]) and deposited into the GenBank database. The un-rooted statistical parsimony network of *Symbiodinium* ITS2 phylotypes found within pocilloporid corals at LHI [53] was updated in TCS v 1.21 (95% connection limit; gaps assigned fifth character state; [66]).

### qPCR analysis of *Symbiodinium* ITS2 ratios

For qPCR analysis, the universal primers CInnerFor (5’-TGGCTTGTTAATTGCTTGGTTCT-3’) and CInnerRev (5’-ACCTGCATCCCAGCGGTT-3’) were developed, in addition to the custom TaqMan fluorogenic probes C100^+^ and C100^−^ (5’-TTTTACTTGAGTGACACCGC-3’ and 5’-CTTTACTTGAGTGACGCTGC-3’, respectively; Life Technologies, Carlsbad, CA, USA). The probe C100^+^ was designed to quantify the number of ITS2 sequences of type C100 in a given sample (denoted C_C100_), while the C100^−^ probe was developed to quantify the copy-number of all clade C ITS2 sequences other than type C100. All primers and probes were initially checked for specificity by conducting a BLAST search against sequences deposited in GenBank [67]. To obtain purified DNA sequences for qPCR calibration, PCR products (types C100, C103, C109 and C118 extracted from *P. damicornis*, and C3 obtained from the Victoria University of Wellington *Symbiodinium* laboratory culture collection) were cloned using the TOPO TA kit (Life Technologies). Plasmid colonies were incubated overnight on selective LB agar plates containing ampicillin, IPTG and XGAL (Bioline). DNA was extracted from positive transformants, purified using a plasmid Mini-Prep kit (Life Technologies), and sequenced as above with the M13 primer set. Plasmid DNA template concentrations were estimated using a Pearl Nanophotometer (Implen, GmbH, Germany), diluted to approximately 10^−3^ ng μl^−1^, and five log_10_ serial dilutions were constructed to generate standard curves and test the accuracy and precision of the assay. All qPCR reactions were carried out in triplicate (standard curves) or duplicate (template solutions) on an Applied Biosystems StepOne instrument (Life Technologies), alongside a template-free control reaction. Each TaqMan qPCR reaction contained 4 μl template, 1× TaqMan Universal Mastermix II (Life Technologies), 1× TaqMan fluorogenic probe (Life Technologies), 18 pmol each primer, and deionised sterile water to a total volume of 20 μl. Thermal cycling conditions involved an initial 10 min, 95°C denaturation step followed by 40 cycles of 15 s at 95°C and 1 min at 60°C. Cycle threshold (C_t_) values were determined as the cycle at which the change in fluorescence was significantly different to the background level (ΔR_n_ = 0.05; obtained using the instrument’s built-in algorithm). C_t_ values below the standard curve intercept (see Additional file 1: Table S1 and Additional file 2: Table S2) and featuring sufficiently low standard deviations (<0.5) were included in the analysis.

To ensure that the TaqMan assays C100^+^ and C100^−^ detected all *Symbiodinium* clade C sequences present within each sample, the total ITS2 copy number (denoted C_TOTAL_) in each *Symbiodinium* cell from the North Bay colonies was also estimated using SYBR qPCR analysis. Reactions were carried out as above, except Power SYBR Green Mastermix (Life Technologies) was used in place of TaqMan Universal Mastermix II, fluorogenic probes were omitted, and C_t_ values were generated using the ΔR_n_ threshold value of 0.3. A melt curve (temperature elevation from 60°C to 95°C in 0.3°C increments each of 15 s duration) was included at the end of each run to ensure that only target sequences were amplified. Template solutions yielding C_t_ values below the standard curve intercept and melting temperatures (T_m_) within 1°C of plasmid T_m_ values were included in the analysis. The ITS2 copy number within each cell (C_TOTAL_; as determined from SYBR qPCR analysis) was compared to the sum of those given by the C100^+^ and C100^−^ TaqMan assays using linear regression (parameters constrained; intercept = 0, slope = 1). Finally, a mixture test was carried out to assess the ability of the TaqMan qPCR assay to predict the proportion of total *Symbiodinium* clade C ITS2 copies that were of type C100 (C_C100_:C_TOTAL_ ratio). Eight mixtures were constructed from plasmid C100 and C109 DNA template solutions (diluted to approximately 200 ITS2 copies μl^−1^; C_C100_:C_TOTAL_ ratios = 0, 0.02, 0.10, 0.4, 0.6, 0.9, 0.98 and 1; see Additional file 3: Table S3 for C_t_ values) and qPCR reactions were carried out in duplicate as above. The ability of the combined TaqMan assay to predict C_TOTAL_ and C_C100_:C_TOTAL_ was assessed using linear regression (parameters constrained; intercept = 0, slope = 1).

### Statistical analysis

**Figure 1:**
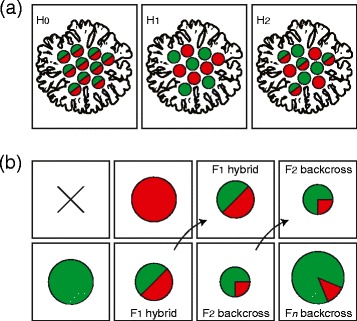
**Conflicting origins of intra-genomic variation in** ***Symbiodinium.*** Red and green colourations represent divergent ITS2 sequences, with monocoloured cells featuring homogeneous ITS2 arrays and bicoloured cells hosting polymorphic ribotypes. Schematics show **(a)** competing hypotheses of sequence homology within *P. damicornis*-associated *Symbiodinium*, with a single clonal population hosting a NDP under H_0_, two genetically isolated populations under H_1_ and inter-lineage recombination under H_2_; and; and **(b)** introgression, with fitness disparities between F_1_ and later generations (shown in a size gradient). Many backcross generations (*n*) may occur before an increase in fitness is realized. Genetic isolation occurs when one or more classes suffer from insurmountably low mean fitness.

To assess the relationship between the total ITS2 copy number and the proportion of copies that were of type C100, a non-linear regression curve (second order polynomial) was fitted to the bivariate C_C100_:C_TOTAL_*versus* C_TOTAL_ data in Sigmaplot v11.0 (Systat, Richmond, CA, USA). Values of C_C100_:C_TOTAL_ were arcsin transformed and compared between colonies (*Colony*) and between branches within colonies (*Branch*(*Colony*)) using nested ANOVA (lm function in R; [68]). Three competing hypotheses were evaluated to explain the ITS2 sequence variation within and between the symbionts of *P. damicornis*: (H_0_) colonies host a single population of genetically heterogeneous symbionts, *versus* (H_1_) colonies host two populations of genetically distinct, homogeneous symbionts, *versus* (H_2_) colonies host distinct populations of genetically homogeneous and heterogeneous symbionts, consistent with the occurrence of recombination (Figure 1a). The proportion of each ITS2 type in the genome of a heterogeneous cell may deviate from codominance (50% each ITS2 type) if recombinants backcross to one or both parental populations (i.e. introgression; Figure 1b). The frequency distribution of C_C100_:C_TOTAL_ within a coral colony (X) is expressed in model form as:

$$ {H}_0:X\sim Beta\left(\alpha, \beta \right),\alpha >1,\beta >1 $$

$$ {H}_1:X\sim Beta\left(\alpha, \beta \right),\alpha <1,\beta <1 $$

$$ {H}_2:X\sim \pi Beta\left({\alpha}_1,{\beta}_1\right)+\left(1\hbox{--} \pi \right) Beta\left({\alpha}_2,{\beta}_2\right),0<\pi <1 $$

where *α* and *β* are the shape parameters of the beta function, and *π* denotes the proportion of symbionts belonging to each component of the mixture model. Mixed beta functions were fitted to the C_C100_:C_TOTAL_ frequency distributions of each coral colony, and maximum likelihood parameter values were solved using the optim function in R (L-BFGS-B method; [68]). A range of starting parameter values was used at each optimization stage to ensure that a universal log-likelihood maximum was reached. Hypothesis evaluation was based on weighted AICc values (*w*_*i*_), with those above 0.90 considered to provide unambiguous support for a candidate model [69,70].

## Results

### DGGE and DNA sequencing

**Figure 2:**
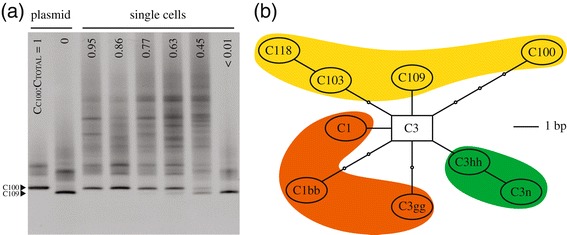
**Sequence variation among pocilloporid-associated** ***Symbiodinium*** **at LHI.** ITS2 sequence variation between and within the *Symbiodinium* genome is shown by: **(a)** DGGE profiles of individual symbionts from *P. damicornis* colonies at Lord Howe Island, featuring a range of C_C100_:C_TOTAL_ ratios (alongside plasmid-purified C100 and C109 DNA); and **(b)** an un-rooted statistical parsimony network showing the phylogenetic relationships between derived pocilloporid-associated *Symbiodinium* types found at Lord Howe Island (ellipses) and the ancestral C3 root (rectangle; modified from [53]). Small circles in **(b)** represent hypothetical intermediate sequences, each distinguished from its neighbour by a single nucleotide substitution or gap. *P. damicornis*-associated types are shown in yellow, while those found in association with *Stylophora pistillata* and *Seriatopora hystrix* are shown in orange and green, respectively.

The excision and sequencing of DGGE bands revealed that all six *P. damicornis* colonies hosted *Symbiodinium* ITS2 types C100 (GenBank accession number HM222433; [53]) and C109 (GenBank accession number KJ530690; novel sequence). No other *Symbiodinium* sequences were detected, including the rare types C103 and C118 previously identified from *P. damicornis* at LHI [53]. While the low resolution and sensitivity of DGGE may have simply precluded their detection, this is unlikely given that this was the same method used in [53]. Alternatively, the absence of C103 and C118 may be explained by differences in host-identification between studies. For example, two ambiguous colonies omitted from the present study that appeared to be the *P. damicornis* × *Stylophora pistillata* hybrids described in [56] were later found to exclusively host *Symbiodinium* C118 (S.P. Wilkinson, unpublished data). Although two divergent ITS2 sequences were retrieved, three distinct DGGE band profiles were observed among the 180 individual cells analysed. These corresponded to *Symbiodinium* cells featuring a homogeneous C109 array, those featuring a near-homogeneous C100 array (with trace copies of C109; hereafter referred to as homogeneous C100), and those with a co-dominant mixture of both ITS2 types (Figure 2). Four of the six colonies analysed hosted a consortium of *Symbiodinium* cells that included all three profiles (two colonies from each site), while the remaining two colonies hosted only homogeneous C100 symbionts and those producing the heterogeneous band-pattern (Figure 2a). No amplification signal was detected from template-free controls or the extractions with symbiont cells omitted, indicating an absence of extracellular DNA contamination.

### qPCR estimation of intra-genomic ITS2 ratios

The universal primers CInnerFor and CInnerRev were identically matched to conserved regions within the ITS2 of *Symbiodinium* C100 and C109. These primers also share identical sequences or single-base pair mismatches with nearly all clade C sequences currently available in the GenBank database, including those found within the corals of LHI [53]. A sequence BLAST analysis of the target probe C100^+^ revealed a high specificity for *Symbiodinium* C100, with at least two nucleotide substitutions differentiating it from the majority of other clade C sequences in GenBank (positioned 16 and 18 base pairs from the 5’ end of the probe). The cytosine at the 5’ end of the probe C100^−^ is mismatched to C100, C109 and the majority of other clade C *Symbiodinium* types (including the ancestral types C1 and C3). This mismatch had no effect on the reaction efficiency when tested on ITS2 types C109 and C103 (95% < E < 100%; see Additional file 1: Table S1 and Additional file 2: Table S2); however it served to prevent cross-hybridization with the C100 sequence. With this exception, C100^−^ shared an identical sequence to most clade C *Symbiodinium* types available in GenBank, including the ancestral types C1, C3 and all derived types found in association with *P. damicornis* at LHI [53]. Standard curve analysis of both TaqMan assays revealed acceptable reaction efficiencies when matched to their respective target sequences (C100^+^ to C100; C100^−^ to both C109 and C103; 95% < E < 100% and R^2^ > 0.99 in all cases; see Additional file 1: Table S1 and Additional file 2: Table S2). qPCR analysis of known plasmid DNA mixtures yielded high accuracy and precision in estimating C_C100_:C_TOTAL_ (constrained linear regression; R^2^ = 0.998; Additional file 3: Table S3, Additional file 4: Figure S1a) and an absence of cross-hybridization. TaqMan qPCR-generated C_TOTAL_ values within each *Symbiodinium* cell were highly correlated with, and not significantly different from those obtained from the SYBR qPCR assay (constrained linear regression; R^2^ = 0.978; Additional file 4: Figure S1b), indicating a negligible incidence of clade C ITS2 types other than those detected by C100^+^ and C100^−^. SYBR qPCR melt curve analysis showed no T_m_ differences between plasmid C100 and C109, and all single-cell templates yielded single T_m_ peaks within 1°C of the plasmid-generated values.

**Figure 3:**
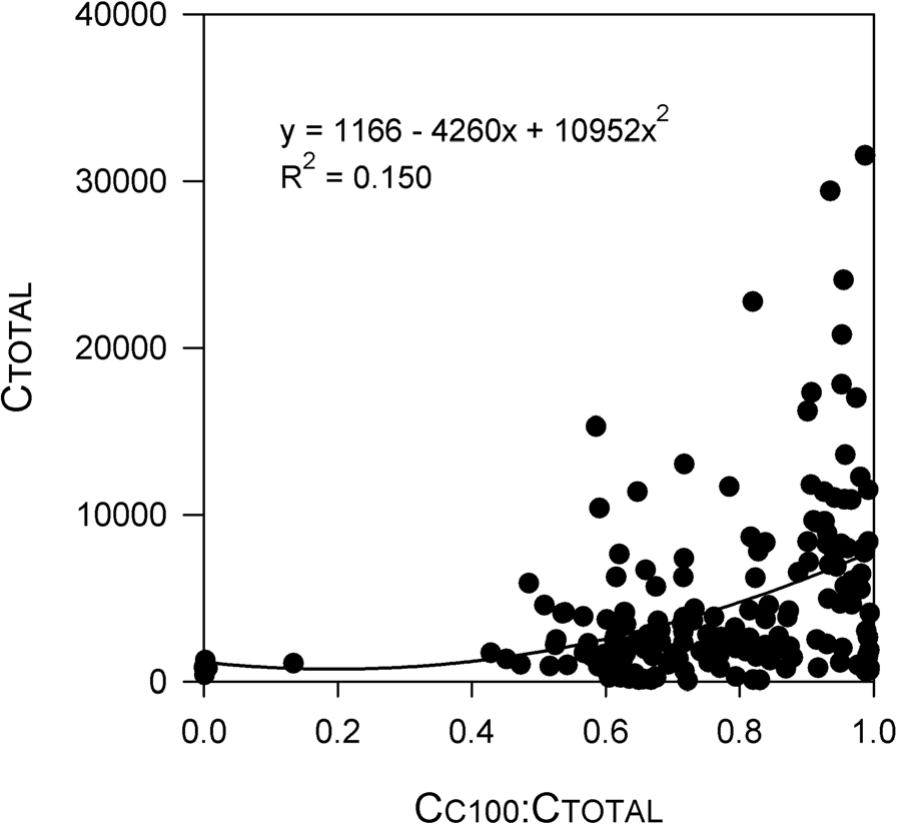
**Variation in ITS2 copy numbers within** ***Symbiodinium*** **cells.** A non-linear relationship existed between the proportion of ITS2 copies of type C100 (C_C100_:C_TOTAL_) and the total number of ITS2 copies within the cell (C_TOTAL_). Homogeneous C100 cells hosted significantly more ITS2 copies than the genetically heterogeneous cells, or those featuring a homogeneous C109 array (second order polynomial regression, p = 0.027).

Within-cell ITS2 copy numbers (C_TOTAL_) ranged from less than 500 to over 30,000, and C_C100_:C_TOTAL_ ratios ranged between 0 and 0.987 (Figure 3; see Additional file 5: Table S4, Additional file 6: Table S5, Additional file 7: Table S6, Additional file 8: Table S7, Additional file 9: Table S8 and Additional file 10: Table S9 for C_t_ values). The remaining ITS2 copies appeared to be primarily of type C109, since this was the only other sequence detected in the DGGE analysis. DGGE band intensities generally reflected qPCR-generated C_TOTAL_ values, and in cases where both C100- and C109-diagnostic bands were present, their relative intensity gave a qualitative indication of C_C100_:C_TOTAL_. However, the C109 band was generally very faint in cells featuring C_C100_:C_TOTAL_ ratios greater than 0.75, and universally undetectable in those above 0.85 (Figure 2a; see Additional file 5: Table S4, Additional file 6: Table S5, Additional file 7: Table S6, Additional file 8: Table S7, Additional file 9: Table S8 and Additional file 10: Table S9). A significant nonlinear correlation between C_C100_:C_TOTAL_ and C_TOTAL_ revealed that ITS2 copy numbers were higher on average in genetically homogeneous C100 cells than in either the heterogeneous C100/C109 cells or the homogeneous C109 cells (non-linear regression, p < 0.027; R^2^ = 0.15; Figure 3). Within-cell C_C100_:C_TOTAL_ ratios did not differ between branches within colonies, but varied between colonies (nested ANOVA, p = 0.82 and < 0.01 for *Branch *(*Colony*) and *Colony* effects, respectively; Table 1).

**Table 1:** **Nested ANOVA output for intra-genomic variation in ITS2 ratios**

**Source of variation**	**df**	**SS**	**MS**	**F**	**P**
**Between colonies**	5	4.07	0.81	24.23	0.001
**Between branches within colonies**	12	0.40	0.03	0.61	0.82
**Error**	162	8.87	0.05		

The model design used in the nested ANOVA analysis was C_C100_:C_TOTAL_ ~ *Colony* + *Branch*(*Colony*). C_C100_:C_TOTAL_ ratios were arcsin transformed prior to analysis. Branches within colonies were pooled for subsequent mixture model fitting.

**Figure 4:**
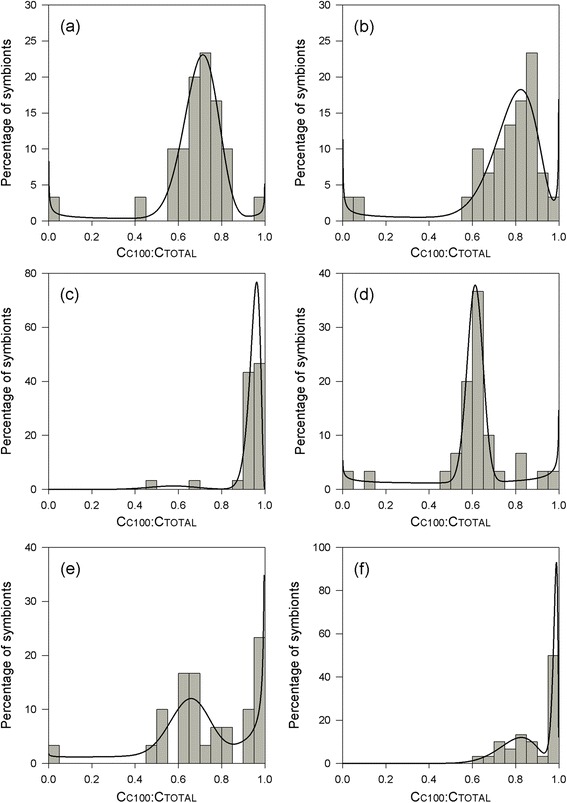
**Frequency distributions of intra-genomic ITS2 ratios in coral colonies.** Colonies **a-c** were sampled from North Bay (1-3 m), and colonies **d-f** from Ned’s Beach (14-18 m). Vertical bars represent the percentage of symbiont cells within each C_C100_: C_TOTAL_ category (*n* = 30 cells for each colony). Overlying probability density functions are optimized two-component beta mixtures (see Table 2 for parameter estimates).

The application and evaluation of competing beta models based on C_C100_:C_TOTAL_ ratios revealed the presence of multiple symbiont clusters in all six colonies. In all cases, the two-component beta mixture model representative of H_2_ provided the best fit of the candidate models (*w*_*i*_ > 0.90 for all colonies; Table 2). Three modes were present in colonies a, b, d and e, representing clusters of genetically homogeneous C100 cells, homogeneous C109 cells, and heterogeneous C100/C109 cells. Two modes were detected in colonies c and f, representing coexisting populations of homogeneous C100 cells and heterogeneous C100/C109 cells (Figure 4). The proportion of genetically heterogeneous symbiont cells in the consortium ranged from 7% in colony c to 88.5% in colony a.

**Table 2:** **Summary of optimized beta mixture models**

**Colony ID**	**Sample site**	**Best-fit hypothesis**	**Model equation**	**Proportion of heterogeneous cells**	**Akaike weight (** ***w***_***i***_ **)**
**a**	North Bay	H_2_	*X* ~ 0.13 × *Beta* (0.57, 0.53) + 0.87 × *Beta* (26.08, 10.89)	0.885	> 0.99
**b**	North Bay	H_2_	*X* ~ 0.07 × *Beta* (3.48, 66.21) + 0.93 × *Beta* (10.99, 2.89)	0.816	> 0.99
**c**	North Bay	H_2_	*X* ~ 0.07 × *Beta* (13.97, 10.32) + 0.93 × *Beta* (57.10, 3.12)	0.007	> 0.99
**d**	Ned’s Beach	H_2_	*X* ~ 0.33 × *Beta* (0.71, 0.57) + 0.67 × *Beta* (108.55, 68.91)	0.670	> 0.99
**e**	Ned’s Beach	H_2_	*X* ~ 0.51 × *Beta* (16.58, 4.28) + 0.49 × *Beta* (114.8, 2.32)	0.512	0.96
**f**	Ned’s Beach	H_2_	*X* ~ 0.55 × *Beta* (0.90, 0.34) + 0.45 × *Beta *(19.40, 10.63)	0.444	0.93

Model support is indicated by Akaike weights (*w*_*i*_), representing the conditional probability that a particular model provides the best fit of all candidate models (i.e. H_0_, H_1_ and H_2_). These give unambiguous support for a candidate if > 0.9 [69,70].

## Discussion

Assessing the incidence of recombination between divergent *Symbiodinium* lineages is made difficult by their apparent haplontic life cycle, a lack of amenability to culture in many types (particularly in clade C *Symbiodinium*), and the paucity of high-resolution single-copy genetic markers. This study attempts to circumvent these obstacles by developing protocols to isolate and extract DNA from individual *Symbiodinium* cells, establish and quantify the dominant ribotype(s) within each genome, and test competing hypotheses explaining the observed pattern of intra-genomic variation. Using these techniques, a population of putative inter-lineage recombinants is identified inhabiting the reef building coral *Pocillopora damicornis* at the isolated, high-latitude reef of Lord Howe Island, Australia.

### Method development

The single-cell isolation and extraction method described here facilitated the rapid preparation of individual *Symbiodinium* cells prior to PCR (around 20 per hour), with the potential to be further improved with the application of flow-cytometry and fluorescence activated cell sorting (FACS). The protocol also showed good efficiency, with around 85% of isolated cells undergoing successful PCR amplification. The downstream application of DGGE and DNA sequencing successfully revealed the dominant ribotype(s) within individual cells, providing a reliable assessment of inter-genomic ITS2 diversity within the *P. damicornis* symbiont consortium. Used in conjunction with plasmid cloning, this method could be used to evaluate levels of intra-genomic variation in other genetic markers, providing an important assessment of their phylogenetic utility.

The qPCR assay developed in this study offers sufficient sensitivity to quantify ITS2 ratios at the sub-clade level. This represents a significant improvement in resolution from earlier clade-level assays [71-76], since the sub-clade presents a more ecologically-relevant taxonomic unit [19]. This assay is also the first to quantify polymorphic rDNA sequences within individual *Symbiodinium* cells, and the second to do so in dinoflagellates (see also [51]). This provides an important insight into the level of ITS2 variation within the *Symbiodinium* genome, underscoring concerns about its utility in establishing diversity estimates [46], and its suitability for quantifying the dynamics of mixed infections [74]. In particular, substantial differences in rDNA copy numbers observed between *Symbiodinium* types C100 and C109 highlight the perils of using ITS2-qPCR to estimate abundance ratios of coexisting symbionts without single-cell validation. Finally, the statistical methodology developed here can identify potential admixture in symbiont populations based on intra-genomic ITS2 ratios. Conflicting hypotheses of one, two and three coexisting populations were formulated, corresponding to the existence of a single symbiont clone harbouring a non-diagnostic polymorphism (NDP), the coexistence of two ‘pure’ (homogeneous) ribotypes, and mixed populations of genetically homogeneous and heterogeneous *Symbiodinium* cells, respectively. The model consistent with the latter hypothesis received unambiguous statistical support in all six *P. damicornis* colonies analysed. However, the model selection approach relies on forming a set of candidate models that are representative of the biological processes under investigation [70]. While the mixture model representing H_2_ is consistent with a population of recombinant genotypes coexisting with parental populations (progenitors), it cannot explicitly prove this scenario. This is because a similar pattern could arise from the incomplete concerted evolution of ancestral polymorphisms (ICEAP).

### Recombination or incomplete concerted evolution of ancestral polymorphisms?

The existence of both C100 and C109 ribotypes in the homogeneous condition affirms their status as diagnostic of separate *Symbiodinium* sub-clades (i.e. neither sequence represents a degenerating pseudo-gene). Furthermore, these two ribotypes differ at five variable nucleotide sites in the ITS2 region (2% divergence), while NDPs typically feature a single nucleotide substitution or insertion/deletion (indel) that distinguishes them from the dominant sequence variant [19,36]. However, if both ribotypes were present within the genome of the most recent common ancestor of *Symbiodinium* C100 and C109, processes of concerted evolution may not have had sufficient time to homogenize the rDNA arrays of both taxa. Hence copies of the ribotype that is now diagnostic of the sister taxon may remain in the genome of one or both lineages. The *Symbiodinium* genome routinely hosts a diverse assemblage of ITS2 sequences [46], and several putative cases of ICEAP appear in the literature. For example, the ITS2 sequence diagnostic of *Symbiodinium glynni* (type D1) also occurs within the genome of *S. trenchii* (type D1a), with the incomplete displacement of a vestigial polymorphism invoked to explain their intra-genomic coexistence [46]. However, several features of the data presented here suggest that an alternative explanation of recombination is feasible. First, the C100 and C109 sequences coalesce at the ancestral type C3, as opposed to either representing an intermediate evolutionary step toward the other (e.g. C103 and C118 in *P. damicornis* and C3hh and C3n in *Seriatopora hystrix*; see Figure 2b). If concerted evolution has not had sufficient time to homogenize all C109 rDNA repeats in the C100 genome, then vestigial copies of the intermediate C3 sequence would also likely persist as a non-dominant intra-genomic variant. Rather, the C3 sequence was not detected in any of the cells analysed, despite its characteristic DGGE band pattern (see supplementary material in [53]). Second, concerted evolutionary processes rapidly homogenize intra-genomic co-dominance, either completely displacing a non-dominant polymorphism or leaving only background traces [44,77,78]. This is inconsistent with the similar proportional abundance of ITS2 polymorphisms within many of the genetically heterogeneous cells observed here, with more than a third of all symbionts featuring C_C100_:C_TOTAL_ ratios of between 0.25 and 0.75. Finally, frequency ‘dips’ along the C_C100_:C_TOTAL_ spectrum depict a degree of genetic isolation between genetically heterogeneous *Symbiodinium* cells and either of the ‘pure’ genotypes (i.e. homogeneous C100 and C109 cells), consistent with the substantial fitness loss often experienced by F_2_ and later-generation backcross genotypes (as a result of processes such as ‘hybrid breakdown’; see [79,80]).

While recombination represents a plausible explanation for the intra-genomic codominance of the C100 and C109 ribotypes, there remains a possibility that this pattern resulted from ICEAP. Addressing this question will likely require a significant investment of resources, including the development of a suite of single-copy markers, the generation of isoclonal cultures or the application of whole genome amplification (WGA; in order to facilitate multi-locus genotyping analysis on individual cells), and/or continued attempts to induce the sexual life cycle, both within and between cultured *Symbiodinium* lineages. Another area requiring investigation is the morphological, physiological and ecological characterization of putative *Symbiodinium* recombinants. Concerted evolution operates *via* a series of stochastic processes that occur independently of natural selection [81]. By contrast, recombination between lineages is often accompanied by drastic changes in morphology, performance and fitness [79,82-84], even involving diversification into new habitats [85]. Investigating the form, function, distribution and ecology of genetically heterogeneous *Symbiodinium* cells may therefore provide further insight into the incidence and potential evolutionary effects of recombination within and between *Symbiodinium* lineages.

### Background symbiont populations

The results of this study indicate that at least three ITS2 genotypes can coexist within the symbiont consortium of *P. damicornis* (C100, C100/C109 and C109). While homogeneous *Symbiodinium* C109 cells were only ever detected at background levels (constituting less than 7% of the symbiont population), the biological relevance of this population may extend well beyond providing a presumably minor contribution to the overall productivity of the symbiosis. Genetically heterogeneous *Symbiodinium* cells outnumbered ‘pure’ genotypes in more than half of the colonies sampled, suggesting that rare sexual reproduction events between C100 and C109 may facilitate asexual proliferation of the F_1_ generation, with potentially important functional implications for the coral colony. The evolutionary contribution of rare *Symbiodinium* types may be more important still, if recombinants create a ‘bridge’ for the migration of genetic material to the dominant lineage (i.e. introgression; see Figure 1b). A small number of genetically heterogeneous symbionts featured C_C100_:C_TOTAL_ ratios near 0.75, and thus potentially represent F_1_ × C100 backcross genotypes. However, this pattern could equally have arisen from ICEAP, differential rDNA inheritance in the F_1_ generation (arising from dissimilar copy-numbers between parent taxa; e.g. [51]), or even concerted evolution acting to homogenize rDNA variability in the recombinant genome (e.g. [77]). Establishing the incidence of introgression would initially require the identification of individual F_1_- and backcross classes. This in turn requires the genotyping of a large number of individuals, and the analysis of at least 13–50 ancestry-informative loci per individual [86,87]. This study was not sufficiently resourced to carry out such a comprehensive task; however it does serve to highlight the perils of dismissing symbionts that persist in low abundance as biologically-irrelevant or simply representing surface contamination.

## Conclusion

While the results presented in this study do not provide unequivocal evidence of recombination between divergent *Symbiodinium* lineages, they provide an initial ‘proof of principle’ for its occurrence. In doing so, this study draws attention to the important evolutionary implications that may accompany the generation of new genetic diversity in *Symbiodinium*, including the potential for rapid symbiont adaptation through introgression. Progress in this area has been hindered by a lack of available methodology, an obstacle that is addressed here through the development of new molecular and statistical methods focused on the individual *Symbiodinium* cell. Additional development of this research may help to characterize and predict the evolutionary response of the coral-algal symbiosis to the many anthropogenic impacts currently threatening the world’s coral reefs.

### Data accessibility

Amino acid sequence data is deposited in GenBank (accession number KJ530690)

Quantitative PCR (qPCR) cycling threshold values and model parameters accompany the manuscript as supplemental information.

## Additional files

Additional file 1: Table S1. Standard curve analysis for nested qPCR (North Bay colonies). Additional file 2: Table S2. Standard curve analysis for nested qPCR (Ned’s Beach colonies). Additional file 3: Table S3. Assay validation for TaqMan nested qPCR. Additional file 4: Figure S1. Single-cell qPCR assay validation. Additional file 5: Table S4. Mean C_t_ values for individual *Symbiodinium* cells (colony a). Additional file 6: Table S5. Mean C_t_ values for individual *Symbiodinium* cells (colony b). Additional file 7: Table S6. Mean C_t_ values for individual *Symbiodinium* cells (colony c). Additional file 8: Table S7. Mean C_t_ values for individual *Symbiodinium* cells (colony d). Additional file 9: Table S8. Mean C_t_ values for individual *Symbiodinium* cells (colony e). Additional file 10: Table S9. Mean C_t_ values for individual *Symbiodinium* cells (colony f).

## References

[1] Davy SK, Allemand D, Weis VM (2012). Cell biology of cnidarian-dinoflagellate symbiosis. Microbiol Mol Biol Rev.

[2] Pochon X, Gates RD (2010). A new *Symbiodinium* clade (Dinophyceae) from soritid foraminifera in Hawai’i. Mol Phylogenet Evol.

[3] Rowan R (2004). Thermal adaptation in reef coral symbiosis. Nature.

[4] Sampayo EM, Franceschinis L, Hoegh-Guldberg O, Dove S (2007). Niche partitioning of closely related symbiotic dinoflagellates. Mol Ecol.

[5] Sampayo EM, Ridgway T, Bongaerts P, Hoegh-Guldberg O (2008). Bleaching susceptibility and mortality of corals are determined by fine-scale differences in symbiont type. Proc Natl Acad Sci U S A.

[6] Berkelmans R, van Oppen MJH (2006). The role of zooxanthellae in the thermal tolerance of corals: a “nugget of hope” for coral reefs in an era of climate change. Proc R Soc B.

[7] Hughes TP, Baird AH, Bellwood DR, Card M, Connolly SR, Folke C (2003). Climate change, human impacts, and the resilience of coral reefs. Science.

[8] Hoegh-Guldberg O, Mumby PJ, Hooten AJ, Steneck RS, Greenfield P, Gomez E (2007). Coral reefs under rapid climate change and ocean acidification. Science.

[9] Carpenter KE, Abrar M, Aeby G, Aronson RB, Banks S, Bruckner A (2008). One-third of reef-building corals face elevated extinction risk from climate change and local impacts. Science.

[10] Baker AC, Starger CJ, McClanahan TR, Glynn PW (2004). Corals’ adaptive response to climate change. Nature.

[11] Coffroth MA, Poland DM, Petrou EL, Brazeau DA, Holmberg JC (2010). Environmental symbiont acquisition may not be the solution to warming seas for reef-building corals. PLoS One.

[12] Rowan R, Knowlton N, Baker A, Jara J (1997). Landscape ecology of algal symbionts creates variation in episodes of coral bleaching. Nature.

[13] Baker AC (2001). Reef corals bleach to survive change. Nature.

[14] Buddemeier RW, Fautin DG (1993). Coral bleaching as an adaptive mechanism. Bioscience.

[15] Stat M, Gates RD (2011). Clade D *Symbiodinium* in scleractinian corals: A “nugget” of hope, a selfish opportunist, an ominous sign, or all of the above?. J Mar Biol.

[16] Stat M, Carter D, Hoegh-Guldberg O (2006). The evolutionary history of *Symbiodinium* and scleractinian hosts— Symbiosis, diversity, and the effect of climate change. Perspect Plant Ecol Evol Syst.

[17] Putnam HM, Stat M, Pochon X, Gates RD (2012). Endosymbiotic flexibility associates with environmental sensitivity in scleractinian corals. Proc R Soc B.

[18] Kirtman B, Power SB, Adedoyin JA, Boer GJ, Bojariu R, Camilloni I, Stocker TF, Qin D, Plattner G-K, Tignor M, Allen SK, Boschung J, Nauels A, Xia Y, Bex V, Midgley P (2013). Near-term climate change: projections and predictability. Clim Chang 2013 Phys Sci Basis Contrib Work Gr I to Fifth Assess Rep Intergov Panel Clim Chang.

[19] LaJeunesse TC (2005). “Species” radiations of symbiotic dinoflagellates in the Atlantic and Indo-Pacific since the Miocene-Pliocene transition. Mol Biol Evol.

[20] Correa AMS, Baker AC (2009). Understanding diversity in coral-algal symbiosis: a cluster-based approach to interpreting fine-scale genetic variation in the genus *Symbiodinium*. Coral Reefs.

[21] van Oppen MJH, Souter P, Howells EJ, Heyward A, Berkelmans R (2011). Novel genetic diversity through somatic mutations: fuel for adaptation of reef corals?. Diversity.

[22] Baillie BK, Belda-Baillie CA, Silvestre V, Sison M, Gomez AV, Gomez ED (2000). Genetic variation in *Symbiodinium* isolates from giant clams based on random-amplified-polymorphic DNA (RAPD) patterns. Mar Biol.

[23] Baillie BK, Monje V, Silvestre V, Sison M, Belda-Baillie CA (1998). Allozyme electrophoresis as a tool for distinguishing different zooxanthellae symbiotic with giant clams. Proc R Soc London B.

[24] LaJeunesse TC (2001). Investigating the biodiversity, ecology, and phylogeny of endosymbiotic dinoflagellates in the genus *Symbiodinium* using the ITS region: in search of a “species” level marker. J Phycol.

[25] Tibayrenc M, Kjellberg F, Arnaud J, Oury B, Frédérique Brenière S, Dardé M-L (1991). Are eukaryotic microorganisms clonal or sexual? A population genetics vantage. Proc Natl Acad Sci U S A.

[26] Santos SR, Gutiérrez-Rodríguez C, Lasker HR, Coffroth MA (2003). *Symbiodinium* sp. associations in the gorgonian *Pseudopterogorgia elisabethae* in the Bahamas: high levels of genetic variability and population structure in symbiotic dinoflagellates. Mar Biol.

[27] Thornhill DJ, Lewis AM, Wham DC, LaJeunesse TC (2013). Host-specialist lineages dominate the adaptive radiation of reef coral endosymbionts. Evolution.

[28] Pettay DT, Wham DC, Pinzón JH, LaJeunesse TC (2011). Genotypic diversity and spatial-temporal distribution of *Symbiodinium* clones in an abundant reef coral. Mol Ecol.

[29] Baums IB, Devlin-Durante MK, LaJeunesse TC (2014). New insights into the dynamics between reef corals and their associated dinoflagellate endosymbionts from population genetic studies. Mol Ecol.

[30] LaJeunesse TC, Wham DC, Pettay DT, Parkinson JE, Keshavmurthy S, Chen CA (2014). Ecologically differentiated stress-tolerant endosymbionts in the dinoflagellate genus *Symbiodinium* (Dinophyceae) Clade D are different species. Phycologia.

[31] Chi J, Parrow MW, Dunthorn M (2014). Cryptic sex in *Symbiodinium* (Alveolata, Dinoflagellata) is supported by an inventory of meiotic genes. J Eukaryot Microbiol.

[32] Mieog JC, van Oppen MJH, Berkelmans R, Stam WT, Olsen JL (2009). Quantification of algal endosymbionts (*Symbiodinium*) in coral tissue using real-time PCR. Mol Ecol Resour.

[33] Santos SR, Gutierrez-Rodriguez C, Coffroth MA (2003). Phylogenetic identification of symbiotic dinoflagellates via length heteroplasmy in domain V of chloroplast large subunit (cp23S)–ribosomal DNA sequences. Mar Biotechnol.

[34] Takabayashi M, Santos SR, Cook CB (2004). Mitochondrial DNA phylogeny of the symbiotic dinoflagellates (*Symbiodinium*, Dinophyta). J Phycol.

[35] Moore RB (2003). Highly organized structure in the non-coding region of the *psbA* minicircle from clade C *Symbiodinium*. Int J Syst Evol Microbiol.

[36] Tonk L, Bongaerts P, Sampayo EM, Hoegh-Guldberg O (2013). SymbioGBR: a web-based database of *Symbiodinium* associated with cnidarian hosts on the Great Barrier Reef. BMC Ecol.

[37] van Oppen MJH, Gates RD (2006). Conservation genetics and the resilience of reef-building corals. Mol Ecol.

[38] Stern RF, Andersen RA, Jameson I, Küpper FC, Coffroth M-A, Vaulot D (2012). Evaluating the ribosomal internal transcribed spacer (ITS) as a candidate dinoflagellate barcode marker. PLoS One.

[39] Coleman AW (2009). Is there a molecular key to the level of “biological species” in eukaryotes? A DNA guide. Mol Phylogenet Evol.

[40] Song J, Shi L, Li D, Sun Y, Niu Y, Chen Z (2012). Extensive pyrosequencing reveals frequent intra-genomic variations of internal transcribed spacer regions of nuclear ribosomal DNA. PLoS One.

[41] Wolf M, Chen S, Song J, Ankenbrand M, Müller T (2013). Compensatory base changes in ITS2 secondary structures correlate with the biological species concept despite intragenomic variability in ITS2 sequences - a proof of concept. PLoS One.

[42] Chen S, Yao H, Han J, Liu C, Song J, Shi L (2010). Validation of the ITS2 region as a novel DNA barcode for identifying medicinal plant species. PLoS One.

[43] Rybalka N, Wolf M, Andersen RA, Friedl T (2013). Congruence of chloroplast- and nuclear-encoded DNA sequence variations used to assess species boundaries in the soil microalga *Heterococcus* (Stramenopiles, Xanthophyceae). BMC Evol Biol.

[44] Baldwin BG, Sanderson MJ, Porter JM, Wojciechowski MF, Campbell CS, Donoghue MJ (1995). The ITS region of nuclear ribosomal DNA: a valuable source of evidence on angiosperm phylogeny. Ann Missouri Bot Gard.

[45] Álvarez I, Wendel JF (2003). Ribosomal ITS sequences and plant phylogenetic inference. Mol Phylogenet Evol.

[46] Thornhill DJ, LaJeunesse TC, Santos SR (2007). Measuring rDNA diversity in eukaryotic microbial systems: how intragenomic variation, pseudogenes, and PCR artifacts confound biodiversity estimates. Mol Ecol.

[47] Miranda LN, Zhuang Y, Zhang H, Lin S (2012). Phylogenetic analysis guided by intragenomic SSU rDNA polymorphism refines classification of “*Alexandrium tamarense*” species complex. Harmful Algae.

[48] Tengs T, Dahlberg OJ, Shalchian-Tabrizi K, Klaveness D, Rudi K, Delwiche CF (2000). Phylogenetic analyses indicate that the 19’hexanoyloxy-fucoxanthin-containing dinoflagellates have tertiary plastids of haptophyte origin. Mol Biol Evol.

[49] Edvardsen B, Shalchian-Tabrizi K, Jakobsen KS, Medlin LK, Dahl E, Brubak S (2003). Genetic variability and molecular phylogeny of *Dinophysis* species (Dinophyceae) from Norwegian waters inferred from single cell analyses of rDNA. J Phycol.

[50] Stat M, Baker AC, Bourne DG, Correa AMS, Forsman Z, Huggett MJ, Lesser MP (2012). Molecular delineation of species in the coral holobiont. Adv Mar Biol Vol 63.

[51] Brosnahan ML, Kulis DM, Solow AR, Erdner DL, Percy L, Lewis J (2010). Outbreeding lethality between toxic Group I and nontoxic Group III *Alexandrium tamarense* spp. isolates: Predominance of heterotypic encystment and implications for mating interactions and biogeography. Deep Sea Res Part II Top Stud Oceanogr.

[52] Harriott VJ, Harrison PL, Banks SA (1995). The coral communities of Lord Howe Island. Mar Freshw Res.

[53] Wicks LC, Sampayo EM, Gardner JPA, Davy SK (2010). Local endemicity and high diversity characterise high-latitude coral–*Symbiodinium* partnerships. Coral Reefs.

[54] Veron JEN. Corals of the World. Townsville: Australian Institute of Marine Science; 2000.

[55] Combosch DJ, Vollmer SV (2013). Mixed asexual and sexual reproduction in the Indo-Pacific reef coral *Pocillopora damicornis*. Ecol Evol.

[56] Miller KJ, Ayre DJ (2004). The role of sexual and asexual reproduction in structuring high latitude populations of the reef coral *Pocillopora damicornis*. Heredity.

[57] Willis BL, van Oppen MJH, Miller DJ, Vollmer SV, Ayre DJ (2006). The role of hybridization in the evolution of reef corals. Annu Rev Ecol Evol Syst.

[58] LaJeunesse TC, Loh WKW, van Woesik R, Hoegh-Guldberg O, Schmidt GW, Fitt WK (2003). Low symbiont diversity in southern Great Barrier Reef corals, relative to those of the Caribbean. Limnol Oceanogr.

[59] LaJeunesse TC, Bhagooli R, Hidaka M, DeVantier L, Done T, Schmidt GW (2004). Closely related *Symbiodinium* spp. differ in relative dominance in coral reef host communities across environmental, latitudinal and biogeographic gradients. Mar Ecol Prog Ser.

[60] Ulstrup KE, Hill R, van Oppen MJH, Larkum AWD, Ralph PJ (2008). Seasonal variation in the photo-physiology of homogeneous and heterogeneous *Symbiodinium* consortia in two scleractinian corals. Mar Ecol Prog Ser.

[61] Stat M, Loh WKW, Hoegh-Guldberg O, Carter DA (2008). Symbiont acquisition strategy drives host–symbiont associations in the southern Great Barrier Reef. Coral Reefs.

[62] Silverstein RN, Correa AMS, LaJeunesse TC, Baker AC (2011). Novel algal symbiont (*Symbiodinium* spp.) diversity in reef corals of Western Australia. Mar Ecol Prog Ser.

[63] Seutin G, White BN, Boag PT (1991). Preservation of avian blood and tissue samples for DNA analyses. Can J Zool.

[64] LaJeunesse TC (2002). Diversity and community structure of symbiotic dinoflagellates from Caribbean coral reefs. Mar Biol.

[65] Stat M, Pochon X, Cowie ROM, Gates RD (2009). Specificity in communities of *Symbiodinium* in corals from Johnston Atoll. Mar Ecol Prog Ser.

[66] Clement M, Posada D, Crandall KA (2000). TCS: a computer program to estimate gene genealogies. Mol Ecol.

[67] Altschul SF, Gish W, Miller W, Myers EW, Lipman DJ (1990). Basic local alignment search tool. J Mol Biol.

[68] R Development Core Team (2011). R: A language and environment for statistical computing.

[69] Burnham KP, Anderson DR (2002). Model Selection and Multimodel Inference: A Practical Information-Theoretic Approach (2nd Edition).

[70] Johnson JB, Omland KS (2004). Model selection in ecology and evolution. Trends Ecol Evol.

[71] Loram JE, Boonham N, O’Toole P, Trapido-Rosenthal HG, Douglas AE (2007). Molecular quantification of symbiotic dinoflagellate algae of the genus *Symbiodinium*. Biol Bull.

[72] Ulstrup KE, van Oppen MJH (2003). Geographic and habitat partitioning of genetically distinct zooxanthellae (*Symbiodinium*) in *Acropora* corals on the Great Barrier Reef. Mol Ecol.

[73] Cunning R, Glynn PW, Baker AC (2013). Flexible associations between *Pocillopora* corals and *Symbiodinium* limit utility of symbiosis ecology in defining species. Coral Reefs.

[74] Mieog JC, van Oppen MJH, Cantin NE, Stam WT, Olsen JL (2007). Real-time PCR reveals a high incidence of *Symbiodinium* clade D at low levels in four scleractinian corals across the Great Barrier Reef: implications for symbiont shuffling. Coral Reefs.

[75] Yamashita H, Suzuki G, Hayashibara T, Koike K (2010). Do corals select zooxanthellae by alternative discharge?. Mar Biol.

[76] Correa AMS, McDonald MD, Baker AC (2009). Development of clade-specific *Symbiodinium* primers for quantitative PCR (qPCR) and their application to detecting clade *D* symbionts in Caribbean corals. Mar Biol.

[77] Wendel JF, Schnabel A, Seelanan T (1995). Bidirectional interlocus concerted evolution following allopolyploid speciation in cotton (*Gossypium*). Proc Natl Acad Sci U S A.

[78] Ganley ARD, Scott B (2002). Concerted evolution in the ribosomal RNA genes of an *Epichloë* endophyte hybrid: comparison between tandemly arranged rDNA and dispersed 5S rrn genes. Fungal Genet Biol.

[79] Barton NH (2001). The role of hybridization in evolution. Mol Ecol.

[80] Demuth JP, Wade MJ (2005). On the theoretical and empirical framework for studying genetic interactions within and among species. Am Nat.

[81] Dover G (1982). Molecular drive: a cohesive mode of species evolution. Nature.

[82] Barton N, Bengtsson BO (1986). The barrier to genetic exchange between hybridising populations. Heredity.

[83] Arnold ML (2007). Evolution through Genetic Exchange.

[84] Arnold ML, Martin NH (2010). Hybrid fitness across time and habitats. Trends Ecol Evol.

[85] Rieseberg LH, Raymond O, Rosenthal DM, Lai Z, Livingstone K, Nakazato T (2003). Major ecological transitions in wild sunflowers facilitated by hybridization. Science.

[86] Epifanio JM, Phillipp DP (1997). Sources for misclassifying genealogical origins in mixed hybrid populations. J Hered.

[87] Fitzpatrick BM (2012). Estimating ancestry and heterozygosity of hybrids using molecular markers. BMC Evol Biol.

