# Supplementary material for: Intra-genomic variation in symbiotic dinoflagellates: recent divergence or recombination between lineages?
Source: BMC Evol Biol. 2015 Mar 14;15:46. doi: 10.1186/s12862-015-0325-1 (PMC4381663; doi:10.1186/s12862-015-0325-1)
Supplement: Additional file 1: Table S1. — Standard curve analysis for nested qPCR (North Bay colonies). [file 12862_2015_325_MOESM1_ESM.pdf]

Table S1Standard curve analysis for nested qPCR (North Bay colonies)

Assay	Template sequence	Concentration (ng μl^{-1})	Concentration (copies μl^{-1})	Mean C_t	Slope	Intercept	Efficiency
**$C100^+$**	C100	1 x 10^{-3}	212345	7.66	-3.5255	26.472	96.08%
**(TaqMan)**		1 x 10^{-4}	21234	11.12			
		1 x 10^{-5}	2123	14.91			
		1 x 10^{-6}	212	18.34			
		1 x 10^{-7}	21	21.68			
	C103	1 x 10^{-3}	212345	-			
	C109	2.5 x 10^{-3}	530862	-			
	C118	1 x 10^{-3}	212345	-			
	C3	1 x 10^{-3}	212345	-			
**$C100^-$**	C109	2.5 x 10^{-3}	530862	6.14	-3.5296	26.392	96.00%
**(TaqMan)**		2.5 x 10^{-4}	53086	9.66			
		2.5 x 10^{-5}	5309	13.4			
		2.5 x 10^{-6}	531	16.8			
		2.5 x 10^{-7}	53	20.22			
	C103	1 x 10^{-3}	212345	8.33	-3.5305	27.054	95.99%
		1 x 10^{-4}	21234	11.67			
		1 x 10^{-5}	2123	15.45			
		1 x 10^{-6}	212	18.54			
		1 x 10^{-7}	21	22.51			
	C100	1 x 10^{-3}	212345	-			
**SYBR**	C100/C109	1 x 10^{-3}	212345	5.79	-3.4024	23.775	98.37%
		1 x 10^{-4}	21234	8.9			
		1 x 10^{-5}	2123	12.48			
		1 x 10^{-6}	212	15.73			
		1 x 10^{-7}	21	19.38			

Mean cycling threshold (C_t) values were calculated from triplicate reactions. Template solutions were plasmid-purified DNA of known *ITS2* sequences. A C100/C109 mixture with C_{C100}:C_{TOTAL} = 0.5 was used for calibration of the SYBR assay. Dashes represent no-amplification reactions, and show an absence of cross-hybridization. R^2 values exceeded 0.99 in all cases
