# Supplementary material for: Intra-genomic variation in symbiotic dinoflagellates: recent divergence or recombination between lineages?
Source: BMC Evol Biol. 2015 Mar 14;15:46. doi: 10.1186/s12862-015-0325-1 (PMC4381663; doi:10.1186/s12862-015-0325-1)
Supplement: Additional file 2: Table S2. — Standard curve analysis for nested qPCR (Ned’s Beach colonies). [file 12862_2015_325_MOESM2_ESM.pdf]

Table S2 Standard curve analysis for nested qPCR (Ned's Beach colonies)

qPCR assay	Template sequence	Concentration (ng µl^{-1})	Concentration (copies µl^{-1})	Mean C_t	Slope	Intercept	Efficiency
**C100^{+}**	C100	6.67 x 10^{-4}	142,271	7.66	-3.5325	30.057	95.95%
		1 x 10^{-4}	21,234	11.12			
		1 x 10^{-5}	2,123	14.91			
		1 x 10^{-6}	212	18.34			
		1 x 10^{-7}	21	21.68			
	C109	1 x 10^{-3}	212,344	-			
**C100^{-}**	C109	1 x 10^{-3}	212,344	6.14	-3.553	29.866	95.59%
		2.5 x 10^{-4}	53,086	9.66			
		2.5 x 10^{-5}	5,309	13.4			
		2.5 x 10^{-6}	531	16.8			
		2.5 x 10^{-7}	53	20.22			
	C100	6.67 x 10^{-4}	142,271	-			

Mean cycling threshold (C_t) values are calculated from triplicate reactions. Template solutions were plasmid-purified DNA of known *ITS2* sequences (C100 or C109). Dashes represent no-amplification reactions, and show an absence of cross-hybridization. R^2 values exceeded 0.99 in both cases.
