# Supplementary material for: Intra-genomic variation in symbiotic dinoflagellates: recent divergence or recombination between lineages?
Source: BMC Evol Biol. 2015 Mar 14;15:46. doi: 10.1186/s12862-015-0325-1 (PMC4381663; doi:10.1186/s12862-015-0325-1)
Supplement: Additional file 3: Table S3. — Assay validation for TaqMan nested qPCR. [file 12862_2015_325_MOESM3_ESM.pdf]

Table S3 Assay validation for TaqMan nested qPCR

Mixture	C_{C100}:C_{TOTAL} (actual)	Mean C_t ($C100^+$)	Mean C_t ($C100^-$)	C_{TOTAL} (predicted)	C_{C100}:C_{TOTAL} (predicted)
**1**	1	18.45	-	188	1
**2**	0.98	18.44	23.7	195	0.970
**3**	0.9	18.53	21.26	207	0.863
**4**	0.6	19.09	19.54	211	0.586
**5**	0.4	19.67	19.08	203	0.418
**6**	0.1	21.49	18.51	197	0.132
**7**	0.02	24.99	18.38	189	0.014
**8**	0	-	18.4	184	0

Mixtures were generated from plasmid C100 and C109 template solutions diluted to approximately 200 *ITS2* copies µl^{-1}. The assay predicted C_{C100}:C_{TOTAL} with a high degree of accuracy and precision (linear regression with constrained parameters; intercept = 0 and slope = 1; R^2 = 0.998). Dashes represent no-amplification reactions, and show an absence of cross-hybridization.
