# Supplementary material for: Intra-genomic variation in symbiotic dinoflagellates: recent divergence or recombination between lineages?
Source: BMC Evol Biol. 2015 Mar 14;15:46. doi: 10.1186/s12862-015-0325-1 (PMC4381663; doi:10.1186/s12862-015-0325-1)
Supplement: Additional file 4: Figure S1. — Single-cell qPCR assay validation. [file 12862_2015_325_MOESM4_ESM.pdf]

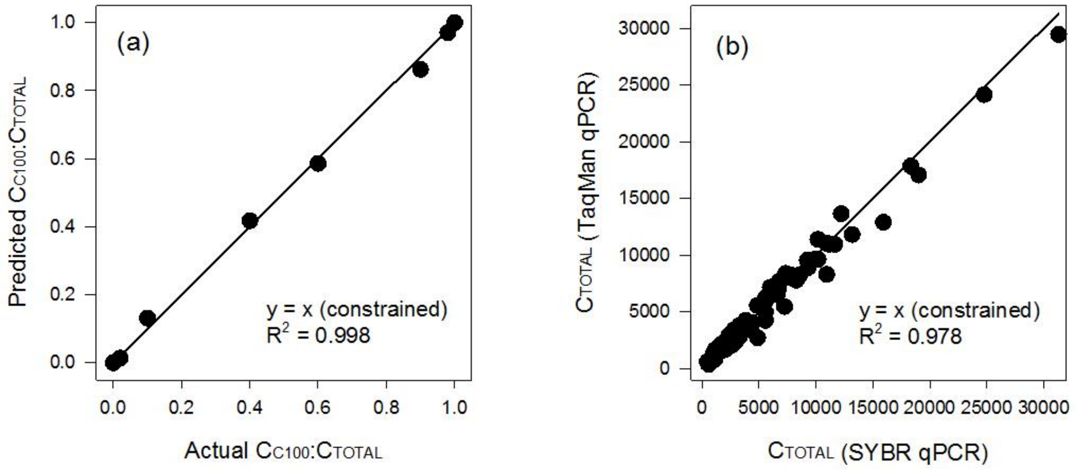

Figure S1 Single-cell qPCR assay validation

The TaqMan qPCR assay was validated by: (a) testing with known mixtures of clonal DNA; and (b) comparing copy-number estimates for each *Symbiodinium* cell (North Bay colonies) with those obtained from the independent SYBR qPCR assay. TaqMan and SYBR qPCR methods gave highly congruent results, implying that the number of *ITS2* copies present within the genome that were not detected by either TaqMan probe was negligible.
