# Supplementary material for: Intra-genomic variation in symbiotic dinoflagellates: recent divergence or recombination between lineages?
Source: BMC Evol Biol. 2015 Mar 14;15:46. doi: 10.1186/s12862-015-0325-1 (PMC4381663; doi:10.1186/s12862-015-0325-1)
Supplement: Additional file 5: Table S4. — Mean Ct values for individual Symbiodinium cells (colony a). [file 12862_2015_325_MOESM5_ESM.pdf]

Table S4 Mean C_t values for individual *Symbiodinium* cells isolated from colony a

Branch	C100 band	C109 band	Mean C_t ($C100^+$)	Mean C_t ($C100^-$)	Mean C_t (SYBR)	C_{TOTAL} (TaqMan)	C_{TOTAL} (SYBR)	C_{C100}:C_{TOTAL}
**1**	Y	Y	19.72	20.89	16.97	2364	1998	0.6945
	Y	N	20.45	24.89	17.89	1077	1072	0.9505
	N	Y	-	21.22	19.26	584	426	0
	Y	Y	19.32	20.73	16.65	2944	2479	0.7278
	Y	Y	19.75	20.69	16.84	2435	2178	0.6616
	Y	Y	21.43	21.69	18.12	970	917	0.5566
	Y	Y	20.52	21.13	17.35	1598	1542	0.6111
	Y	Y	21	22.25	18	1010	995	0.7049
	Y	N	19.37	21.24	16.65	2644	2482	0.7822
	Y	Y	20.69	22.4	17.88	1145	1083	0.7639
**2**	Y	Y	20.24	21.54	17.79	1645	1150	0.7126
	Y	Y	20.95	20.51	17.21	1662	1701	0.4424
	Y	Y	22.34	23.25	18.97	453	516	0.6561
	Y	Y	21.19	23.05	17.86	809	1097	0.7805
	Y	N	19.31	21.58	16.82	2606	2215	0.8233
	Y	Y	19.52	20.56	16.26	2767	3226	0.6758
	Y	Y	19.09	20.49	16.46	3426	2825	0.7254
	Y	Y	18.82	20.6	15.93	3828	4031	0.7721
	Y	Y	21.69	23.12	18.36	625	782	0.7288
	Y	N	19.89	22.1	17.16	1804	1758	0.8179
**3**	Y	Y	17.05	18.48	13.91	12896	15919	0.7292
	Y	Y	19.27	20.42	16.4	3200	2947	0.692
	Y	Y	22.08	22.78	18.5	565	710	0.625
	Y	N	18.97	20.5	16.26	3626	3224	0.7418
	Y	Y	19.3	21.02	16.5	2830	2755	0.7646
	Y	N	19.03	21.09	16.36	3210	3033	0.8025
	Y	Y	20.66	21.15	17.57	1504	1331	0.5924
	Y	Y	20.43	21.52	17.27	1515	1627	0.6826
	Y	Y	19.27	19.49	15.8	4004	4416	0.55
	Y	Y	19.53	20.26	16.73	2948	2360	0.6302

C100- and C109-diagnostic DGGE bands are scored as present or absent (Y or N). Dashes represent no-amplification reactions
