# Supplementary material for: Intra-genomic variation in symbiotic dinoflagellates: recent divergence or recombination between lineages?
Source: BMC Evol Biol. 2015 Mar 14;15:46. doi: 10.1186/s12862-015-0325-1 (PMC4381663; doi:10.1186/s12862-015-0325-1)
Supplement: Additional file 6: Table S5. — Mean Ct values for individual Symbiodinium cells (colony b). [file 12862_2015_325_MOESM6_ESM.pdf]

Table S5 Mean C_t values for individual *Symbiodinium* cells isolated from colony b

Branch	C100 band	C109 band	Mean C_t ($C100^+$)	Mean C_t ($C100^-$)	Mean C_t (SYBR)	C_{TOTAL} (TaqMan)	C_{TOTAL} (SYBR)	C_{C100}:C_{TOTAL}
**1**	Y	Y	20.14	21.99	17.3	1607	1603	0.7798
	Y	Y	18.72	21.25	16.17	3736	3446	0.8466
	Y	N	19.51	21.26	16.45	2459	2852	0.7682
	Y	N	20.13	26.49	17.35	1475	1550	0.9853
	Y	Y	19.99	21	16.87	2057	2138	0.672
	Y	Y	20.01	20.87	16.54	2094	2667	0.6499
	Y	Y	20.38	21.2	17.27	1663	1638	0.6434
	Y	N	19.43	21.45	16.57	2489	2626	0.7977
	Y	Y	18.57	20.84	16.02	4245	3812	0.8232
	Y	N	19.78	22.72	17.31	1800	1588	0.878
**2**	N	Y	-	21.89	18.81	377	577	0
	Y	N	19.18	21.94	15.66	2708	4864	0.8653
	Y	Y	19.7	21.11	16.43	2298	2884	0.7276
	N	Y	25.18	20.74	18.32	848	801	0.0549
	Y	N	18.13	22.15	15.46	4974	5542	0.9361
	Y	N	19.55	22.19	16.98	2149	1987	0.8552
	Y	N	20.11	23.16	17.5	1439	1401	0.8853
	Y	Y	18.2	19.22	15.38	6597	5882	0.6727
	Y	N	20.35	22.96	17.78	1277	1160	0.8528
	Y	N	19.53	21.74	16.96	2282	2017	0.8178
**3**	Y	Y	20.52	21.88	17.75	1354	1178	0.7203
	Y	N	17.5	20.02	14.46	8302	10922	0.8462
	Y	Y	20.52	20.93	16.93	1683	2052	0.5812
	Y	Y	19.29	20.1	15.87	3393	4212	0.6438
	Y	N	20.54	26.28	17.9	985	1066	0.9781
	Y	N	19.55	22.53	17.3	2088	1602	0.8808
	Y	N	19.33	21.03	16.51	2783	2727	0.7626
	Y	Y	18.92	20.34	16.24	3803	3289	0.7279
	Y	N	17.97	20.34	15.47	6187	5535	0.832
	Y	N	17.61	21.04	15.35	7168	5981	0.9082

C100- and C109-diagnostic DGGE bands are scored as present or absent (Y or N). Dashes represent no-amplification reactions
