# Supplementary material for: Intra-genomic variation in symbiotic dinoflagellates: recent divergence or recombination between lineages?
Source: BMC Evol Biol. 2015 Mar 14;15:46. doi: 10.1186/s12862-015-0325-1 (PMC4381663; doi:10.1186/s12862-015-0325-1)
Supplement: Additional file 7: Table S6. — Mean Ct values for individual Symbiodinium cells (colony c). [file 12862_2015_325_MOESM7_ESM.pdf]

Table S6 Mean C_t values for individual *Symbiodinium* cells isolated from colony c

Branch	C100 band	C109 band	Mean C_t ($C100^+$)	Mean C_t ($C100^-$)	Mean C_t (SYBR)	C_{TOTAL} (TaqMan)	C_{TOTAL} (SYBR)	C_{C100}:C_{TOTAL}
**1**	Y	N	16.87	20.74	14.57	11391	10152	0.9299
	Y	N	17.88	23.9	15.67	5557	4834	0.9817
	Y	N	17.36	23.9	14.87	7778	8259	0.9869
	Y	N	16.54	21.34	14.3	13651	12210	0.9603
	Y	N	17.24	21.22	14.7	8895	9301	0.9345
	Y	N	17.6	21.65	15.19	7026	6683	0.9372
	Y	N	15.67	20.37	13.25	24149	24764	0.958
	Y	N	16.18	21.75	13.64	17066	18997	0.9757
	Y	N	17.6	21.96	15.23	6915	6473	0.9478
	Y	N	17.77	22.99	15.44	6048	5620	0.9695
**2**	Y	N	17.93	23.1	15.07	5460	7233	0.9686
	Y	N	16.14	20.73	13.7	17854	18335	0.955
	Y	N	17.12	21.02	14.57	9625	10166	0.931
	Y	N	15.4	19.49	12.91	29461	31310	0.9388
	Y	N	17.16	20.78	14.71	9559	9239	0.9184
	Y	N	17.35	22.28	14.95	8019	7854	0.9635
	Y	N	18.47	21.43	15.46	4231	5547	0.8799
	Y	Y	21.24	20.95	18.07	1305	951	0.4659
	Y	N	17.45	21.7	15.18	7667	6733	0.9445
	Y	N	17.31	23.75	15.01	8034	7554	0.986
**3**	Y	N	16.84	20.33	14.19	11815	13153	0.9116
	Y	N	16.89	21.16	14.46	11056	10918	0.945
	Y	N	17.35	21.32	14.98	8266	7697	0.9338
	Y	Y	19.52	20.56	16.7	2776	2396	0.6758
	Y	N	17.15	20.7	14.59	9661	10009	0.9149
	Y	N	16.87	22.02	14.37	10947	11643	0.9684
	Y	N	17.38	20.77	15.05	8387	7339	0.9066
	Y	N	16.88	21.62	14.43	10956	11144	0.959
	Y	N	17.65	23.72	15.21	6491	6571	0.9824
	Y	N	17.32	21.89	14.82	8276	8585	0.9545

C100- and C109-diagnostic DGGE bands are scored as present or absent (Y or N). Dashes represent no-amplification reactions
