# Supplementary material for: Intra-genomic variation in symbiotic dinoflagellates: recent divergence or recombination between lineages?
Source: BMC Evol Biol. 2015 Mar 14;15:46. doi: 10.1186/s12862-015-0325-1 (PMC4381663; doi:10.1186/s12862-015-0325-1)
Supplement: Additional file 8: Table S7. — Mean Ct values for individual Symbiodinium cells (colony d). [file 12862_2015_325_MOESM8_ESM.pdf]

Table S7 Mean C_t values for individual *Symbiodinium* cells isolated from colony d

Branch	C100 band	C109 band	Mean C_t ($C100^+$)	Mean C_t ($C100^-$)	C_{C100}	C_{TOTAL}	C_{C100}:C_{TOTAL}
**1**	N	Y	27.01	23.92	146	1092	0.1339
	Y	Y	23.26	23.75	1685	2741	0.6147
	Y	Y	23.63	23.56	1324	2515	0.5264
	Y	Y	24.01	24.49	1033	1685	0.6132
	Y	Y	24.64	25	683	1153	0.5925
	Y	Y	25.16	24.78	487	1029	0.4732
	Y	Y	22.58	23.16	2616	4164	0.6282
	Y	Y	25.02	25.36	533	905	0.589
	Y	Y	22.28	23.6	3192	4356	0.7327
	Y	Y	23.65	23.88	1307	2278	0.5737
**2**	Y	N	23.25	30.62	1690	1703	0.9928
	Y	Y	26.5	27.41	203	301	0.6741
	Y	Y	22.76	22.58	2334	4589	0.5086
	Y	N	23.13	25.44	1828	2180	0.8385
	Y	Y	21.98	22.47	3868	6282	0.6158
	Y	Y	20.69	20.98	8968	15307	0.5859
	Y	Y	28.19	28.94	68	104	0.6495
	Y	Y	23.4	23.88	1533	2501	0.613
	Y	Y	27.72	28.52	92	140	0.6565
	Y	Y	22.84	23.02	2208	3898	0.5665
**3**	Y	Y	26.96	27.41	151	250	0.6052
	Y	N	21.19	23.37	6474	7821	0.8278
	Y	Y	24.32	24.89	844	1349	0.6259
	Y	Y	21.67	22.19	4750	7653	0.6207
	Y	Y	25.2	25.73	476	769	0.6191
	Y	Y	26.77	27.69	171	253	0.6753
	Y	Y	27.33	27.98	118	186	0.6354
	Y	N	19.94	23.11	14622	16221	0.9014
	N	Y	32.31	24.13	5	828	0.0056
	Y	Y	21.27	21.6	6145	10400	0.5908

C100- and C109-diagnostic DGGE bands are scored as present or absent (Y or N). Dashes represent no-amplification reactions
