# Supplementary material for: Intra-genomic variation in symbiotic dinoflagellates: recent divergence or recombination between lineages?
Source: BMC Evol Biol. 2015 Mar 14;15:46. doi: 10.1186/s12862-015-0325-1 (PMC4381663; doi:10.1186/s12862-015-0325-1)
Supplement: Additional file 9: Table S8. — Mean Ct values for individual Symbiodinium cells (colony e). [file 12862_2015_325_MOESM9_ESM.pdf]

Table S8 Mean C_t values for individual *Symbiodinium* cells isolated from colony e

Branch	C100 band	C109 band	Mean C_t ($C100^+$)	Mean C_t ($C100^-$)	C_{C100}	C_{TOTAL}	C_{C100}:C_{TOTAL}
**1**	Y	N	20.81	28.04	8320	8386	0.9922
	Y	N	23.74	30.54	1232	1245	0.9896
	Y	N	23.73	25.9	1236	1498	0.825
	Y	Y	23.6	25	1350	1820	0.7418
	Y	Y	23.34	25.19	1599	2013	0.7943
	Y	N	21.36	24.31	5814	6548	0.8878
	Y	N	24.62	27.33	692	796	0.8696
	Y	N	18.78	25.26	31145	31541	0.9875
	Y	N	19.48	23.86	19799	20783	0.9527
	Y	Y	21.75	22.94	4494	6274	0.7163
**2**	Y	Y	20.66	22.41	9175	11692	0.7847
	Y	Y	21.5	22.69	5307	7399	0.7172
	Y	N	21.45	26	5482	5727	0.9572
	Y	N	20.24	26.01	12025	12268	0.9802
	Y	Y	23.67	24.45	1290	1959	0.6585
	Y	N	21.99	24.35	3843	4557	0.8434
	Y	N	22.37	29.11	3010	3042	0.9893
	Y	N	22.58	29.74	2625	2646	0.9918
	Y	N	20.32	27.56	11414	11503	0.9923
	Y	N	27.74	29.88	91	111	0.8204
**3**	Y	N	23.64	30.62	1315	1328	0.9908
	Y	N	24.39	31.05	804	813	0.9885
	Y	N	24.83	31.47	604	611	0.9884
	Y	N	19.83	23.11	15709	17308	0.9076
	Y	N	21.34	26.63	5890	6053	0.973
	Y	Y	21.05	23.12	7092	8681	0.817
	Y	Y	26.72	27.19	176	290	0.6077
	Y	N	21.76	26.76	4479	4629	0.9676
	Y	N	23.07	27.5	1907	2000	0.9535
	Y	N	22.19	24.91	3373	3870	0.8717

C100- and C109-diagnostic DGGE bands are scored as present or absent (Y or N). Dashes represent no-amplification reactions
