# Supplementary material for: Intra-genomic variation in symbiotic dinoflagellates: recent divergence or recombination between lineages?
Source: BMC Evol Biol. 2015 Mar 14;15:46. doi: 10.1186/s12862-015-0325-1 (PMC4381663; doi:10.1186/s12862-015-0325-1)
Supplement: Additional file 10: Table S9. — Mean Ct values for individual Symbiodinium cells (colony f). [file 12862_2015_325_MOESM10_ESM.pdf]

Table S9 Mean C_t values for individual *Symbiodinium* cells isolated from colony f

Branch	C100 band	C109 band	Mean C_t ($C100^+$)	Mean C_t ($C100^-$)	C_{C100}	C_{TOTAL}	C_{C100}:C_{TOTAL}
**1**	Y	N	27.91	30.16	81	98	0.8306
	Y	Y	26.25	28.12	240	302	0.7947
	Y	N	24.37	32.07	815	819	0.9941
	Y	Y	22.59	23.17	2608	4146	0.629
	Y	Y	22.83	22.84	2230	4135	0.5393
	Y	Y	22.68	23.59	2459	3627	0.678
	Y	Y	22.44	22.12	2866	5904	0.4855
	Y	N	21.9	29.65	4075	4098	0.9944
	Y	N	24.68	32.27	666	670	0.9937
	Y	Y	28.9	30.18	43	59	0.7227
**2**	Y	N	21.77	26.08	4450	4684	0.9502
	N	Y	32.94	23.46	3	1274	0.0024
	Y	N	23.09	30.7	1882	1894	0.9938
	Y	Y	20.99	21.69	7375	11389	0.6476
	Y	Y	25.18	25.07	480	929	0.5169
	Y	Y	28.12	29	71	106	0.6678
	Y	Y	26.99	27.55	148	238	0.622
	Y	N	24.51	28	746	813	0.9173
	Y	N	23.56	30.55	1386	1399	0.9908
	Y	Y	22.92	23.87	2096	3073	0.682
**3**	Y	Y	21.99	22.88	3843	5693	0.675
	Y	N	22.93	26.7	2082	2239	0.9303
	Y	N	19.57	21.65	18671	22777	0.8197
	Y	Y	22.88	24.71	2151	2717	0.792
	Y	Y	24.29	25.36	861	1233	0.6982
	Y	Y	25.27	25.73	455	746	0.609
	Y	N	23.4	30.44	1538	1552	0.9911
	Y	Y	23.84	23.76	1154	2201	0.5246
	Y	N	22.78	26.21	2304	2518	0.9151
	Y	Y	22.82	23.23	2237	3717	0.6019

C100- and C109-diagnostic DGGE bands are scored as present or absent (Y or N). Dashes represent no-amplification reactions
